# Photonic Liquid Crystal Polymer Absorbent for Immobilization and Detection of Gaseous Nerve Agent Simulants

**DOI:** 10.1021/acsaom.2c00014

**Published:** 2022-09-06

**Authors:** Yari Foelen, Roberta Puglisi, Michael G. Debije, Albert P. H. J. Schenning

**Affiliations:** † Laboratory of Stimuli-Responsive Functional Materials and Devices, Department of Chemical Engineering and Chemistry, Eindhoven University of Technology, Den Dolech 2, 5600 MB Eindhoven, The Netherlands; ‡ Department of Chemical Sciences, University of Catania, Viale A. Doria 6, 95100 Catania, Italy; § SCNU-TUE Joint Laboratory of Device Integrated Responsive Materials (DIRM), South China Normal University, Guangzhou Higher Education Mega Center, Guangzhou 510006, China; ∥ Institute for Complex Molecular Systems, Eindhoven University of Technology, Den Dolech 2, 5600 MB Eindhoven, The Netherlands

**Keywords:** photonic polymer sensor, optical detection, absorption, nerve agent remediation, cholesteric liquid crystal polymer, phosphoryl group

## Abstract

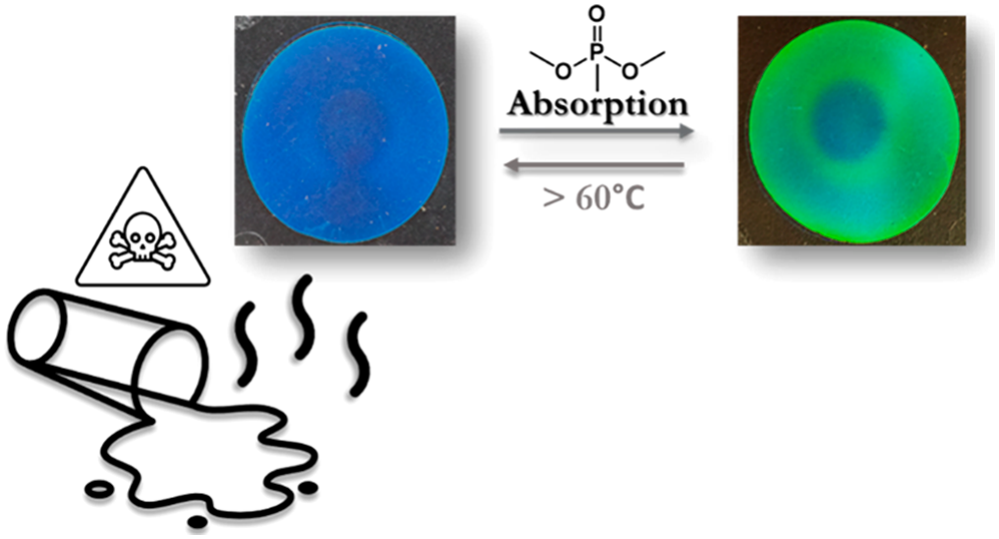

Detection and sequestration of chemical warfare agents (CWAs), such as poisonous organophosphates, are highly desirable for both personal security and environmental protection. However, both sensing and absorption in a single device have been rarely reported. In this study, we describe a photonic absorbent based on a cholesteric liquid crystal polymer as a dual sensing and decontamination device for gas-type CWAs. Dimethyl methylphosphonate (DMMP) was used as a simulant compound. A blue reflective photonic polymer was fabricated that was able to detect DMMP vapor through absorption. Hydrogen bond interactions between DMMP and mesogenic carboxylic groups of the polymer allow selectivity and capture. A distinct optical change of the film from blue to bright green indicates the absorption of DMMP vapor molecules and confirms when full absorption of the polymer is achieved. The diffusion of DMMP vapor into the material was observed by the formation of a sharp boundary between swollen and unswollen material, as evidenced by scanning electron microscopy images and the structural color changes. In ambient conditions, DMMP molecules are retained in the photonic absorbent without release to the environment. Heating above approximately 60 °C releases the absorbed DMMP, leading to a reusable optical device. These results confirm the ability of photonic polymers to sense and immobilize dangerous vapor, paving the way for the realization of simple, battery-free optical devices able to simultaneously warn and protect.

## Introduction

Photonic crystals have attracted much attention as battery-free detection devices, providing clear optical responses including a visible color change.^1−4^ Among photonic crystals, cholesteric liquid crystals (CLCs) have emerged as optical sensors for vapor analytes due to their easy processability.^5,6^ CLCs can be printed or bar coated, and after photopolymerization of the mesogenic mixture, a solid photonic polymer is obtained.^7,8^ CLCs have a periodic helical molecular arrangement due to the presence of a chiral dopant in the liquid crystal mixture. The photonic polymer selectively reflects a specific bandwidth of light determined by the pitch length of the helices and the average refractive index of the polymer. A variety of CLC-based sensors have been reported responding to specific exposures, including humidity,^9,10^ temperature,^11,12^ and solutions containing organic solvents or amines.^13,14^ These CLC films react to the presence of analytes, leading to a change in the helical pitch and consequent blue (shrinkage) or red (swelling) shifts of the selective reflection band (SRB), causing visible color changes of the material.^15^ The color reverts to the original state when equilibrium is restored under ambient conditions. However, photonic polymers have rarely been studied for gas detection,^16,17^ and extended sequestration of gas molecules after exposure has seldom been reported.

Organophosphates are highly toxic compounds widely used as fertilizers, herbicides, pesticides, and as chemical warfare agents (CWAs), also known as nerve agents.^18,19^ Current decontamination procedures normally consist of transport of the CWAs to available sites for harmless degradation. Transport and storage are hazardous as any release could cause fatalities. Development of immobilizing materials able to selectively absorb the toxic agents is a challenging goal.^20−22^ In addition, current identification and detection methods for nerve agents require expensive, unwieldy equipment which are not easy to use in the field.^23−28^

Optical detection methods allow readout using easy-to-use, portable equipment.^29−31^ For example, optical systems based on supramolecular interactions warrant selectivity and reversibility which decreases the risk of false-positive responses.^32−34^ However, most of the existing optical indicators are used for detection of organophosphates in solution.^30^ Furthermore, only by applications based on organogels, simultaneous sequestration and detection of organophosphorus compounds have been reported before.^35,36^

In this work, we present a photonic absorbent based on a CLC polymer for organophosphate gas immobilization, providing both protection and detection simultaneously. The absorption of a Sarin nerve agent simulant,^37^ dimethylmethylphosphonate (DMMP) vapor, is indicated by a color change visible to the naked eye. We demonstrate that the photonic absorber can trap DMMP without releasing it to the environment unless it is heated above 60 °C. The optical response of the polymer to different amounts of DMMP and its selectivity toward phosphoryl oxygen, found in nerve agents and pesticides, reveal that these photonic polymers are attractive as inexpensive, battery-free devices that combine sequestration and detection.

## Results and Discussion

**Figure 1:**
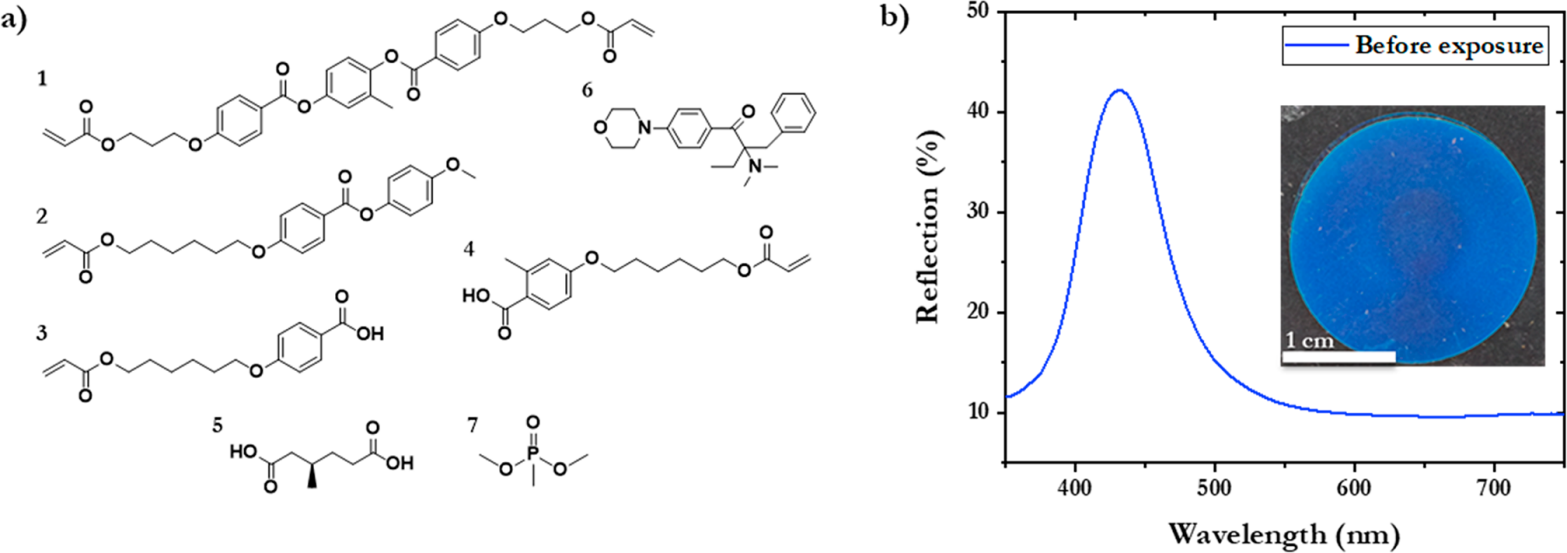
(a) Structures of the chemicals used for the fabrication of the photonic CLC polymer films (**1**–**6**) and structure of DMMP (**7**). (b) UV–vis reflection spectrum of the photonic polymer after evaporation of porogen **5**; inset shows a photograph of the blue structural colored polymer.

**Figure 2:**
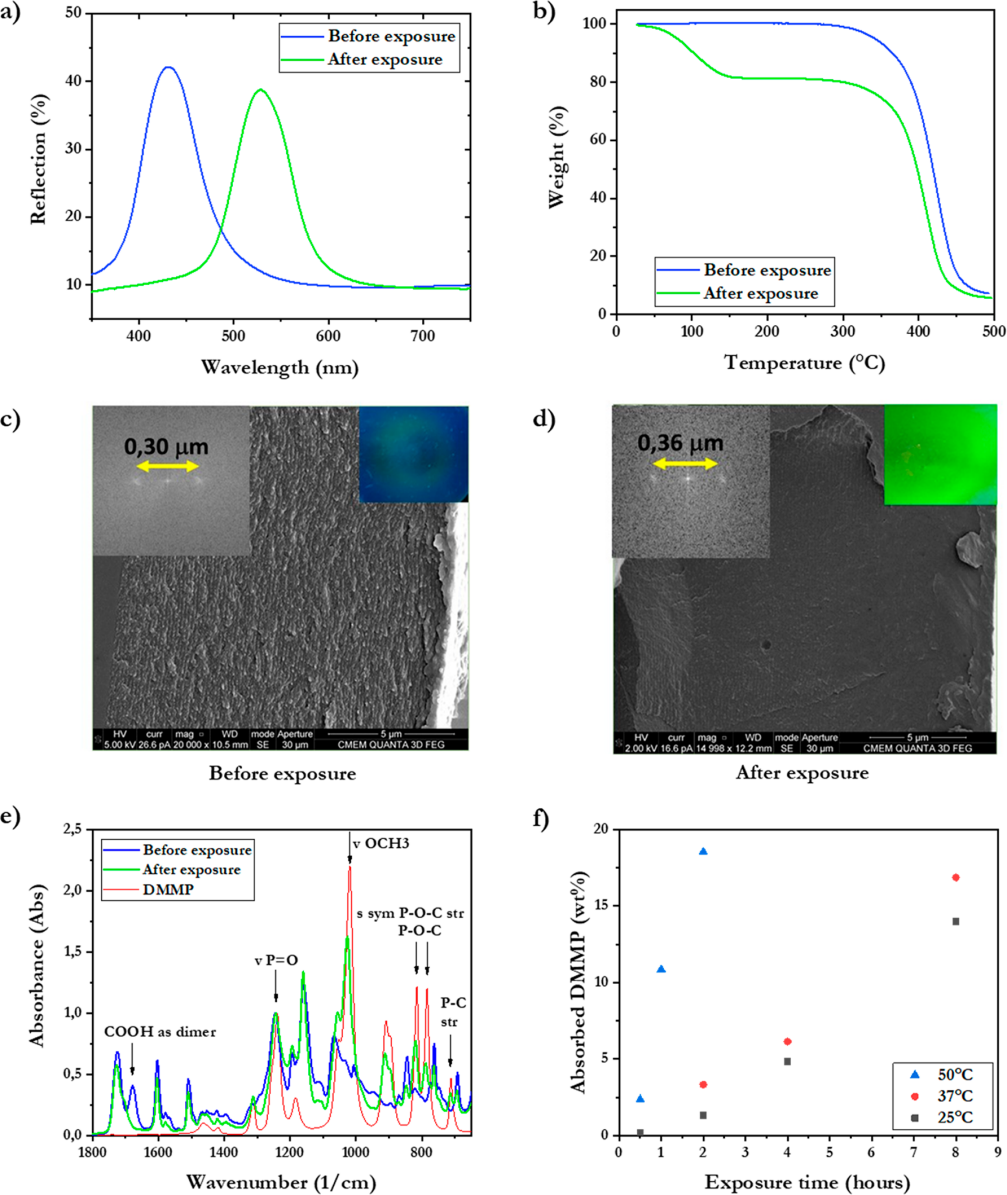
Polymer response before and after exposure to DMMP vapor. (a) UV–vis reflection spectra of the photonic polymer before (blue curve) and after (green curve) exposure. (b) TGA demonstrates weight loss during heating of a pristine sample (blue) and after exposure to DMMP (green). Cross-sectional SEM images of the polymer (c) before and (d) after exposure, with left insets showing a two-dimensional Fourier transformation to illustrate the pitch in the cholesteric order and right insets showing photographs of the polymer. (e) FT-IR spectra before and after exposure displays DMMP complexation through hydrogen bond interaction as the −COOH dimer peak at 1700 cm^–1^ disappears (the spectrum of pure DMMP is added for reference). (f) Temperature influence on the absorption rate of DMMP measured by TGA.

The fabrication of the photonic CLC polymer has been previously reported.^38−40^ The CLC mixture used to prepare the polymer film consists of diacrylate **1** (22.6 wt %) and monoacrylate **2** (26.7 wt %) mesogens. Nonpolymerizable (*R*)-(+)-3-methyladipic acid (MAA) **5** (16.4 wt %) was added as a chiral dopant to induce a CLC phase; **5** also acts as a porogen, creating free volume to obtain a photonic polymer absorbent. Irgacure 369 (**6**, 1.5 wt %) was used as a photoinitiator for UV photopolymerization. Incorporation of benzoic acid-functionalized monoacrylate liquid crystal molecules **3** (16.4 wt %) and **4** (16.4 wt %) provides carboxylic acid end groups for interacting with DMMP (**7**) via a supramolecular hydrogen bond (Figure 1a). The polymer films (20 mg) are produced on glass substrates through shear alignment with a top substrate that is removed after polymerization, exposing one side of the film to air, while the other side remains adhered to the glass substrate. After polymerization, the porogen (**5**) is removed through evaporation in a vacuum oven at 200 °C, and the elastic polymer contracts. Thermogravimetric analysis (TGA) demonstrates the evaporation of the porogen upon heating, corresponding to 18.2 wt % loss of the polymer (Figure S1). The resulting polymer appears bright blue with a corresponding reflection band at ∼430 nm (Figure 1b) and displays thermal degradation above 300 °C (Figure 2b). Differential scanning calorimetry (DSC) indicated a glass transition temperature (*T*_g_) of approximately 60 °C (Figure S2).

To investigate the absorption and optical response, the polymer is exposed to DMMP in a closed vial (0.2 L) in an oven at 50 °C to compensate for the slow evaporation rate of DMMP.^41^ A 10 μL drop is inserted in the vial at a distance from the polymer. This guarantees a sufficient amount of DMMP vapor, as the required volume for vapor saturation in the vial is 5.18 μL at 50 °C (calculated based on vapor pressure data).^42^ After 2 h exposure of the polymer to DMMP vapor, the reflected color shifts from the initial blue (432 nm) to bright green (528 nm) (Figure 2a). Remarkably, after removal from the vial, the polymer retained its green color, indicating the DMMP is strongly absorbed. Scanning electron microscopy (SEM) images of the photonic polymer reveal an increase in the pitch length: based on the two-dimensional Fourier transformation, the pitch length is calculated as 0.30 μm before exposure, corresponding to the blue color (Figure 2c), and 0.36 μm after exposure, consistent with the green color, assuming a refractive index of 1.5 (Figure 2d). The increase in the pitch length of the helical structure of the polymer film is caused by the absorption of DMMP vapor molecules leading to volumetric expansion, inducing elongation of the helical nanostructure and longer reflected wavelengths. Therefore, the quantity of DMMP absorbed is directly correlated to the reflection wavelength of the photonic polymer.

Thermogravimetric analysis shows that the absorbed DMMP in the saturated polymer is retained at ambient conditions and only released by heating the polymer above 60 °C (*T*_g_). The release is completed at approximately 140 °C, consistent with the boiling point of DMMP (bp = 130 °C). Interestingly, the absorbed DMMP is retained in the polymer in ambient conditions for at least 10 weeks (Figure S3). The weight of the polymer film decreases by 18.5 wt % and matches the weight loss after porogen evaporation, indicating that the polymer absorption capacity is determined by the initial amount of porogen (Figure 2b). The weight loss of the released DMMP converts to an absorption capacity of 22.7% of the polymer weight. After longer DMMP vapor exposure of the polymer, or straight contact with liquid DMMP, the retention equilibrium after evaporation of excess DMMP was again 18.5 wt %, indicating the saturation limit.

Fourier transform infrared (FT-IR) analysis shows that the absorption and retention of DMMP is enabled by the hydrogen bonding sites of the polymer (Figure 2e): a sharp vibration peak at 1690 cm^–1^ was observed in the polymer before exposure, confirming the presence of dimeric H-bond interactions provided by the benzoic acid mesogens **3** and **4**. After exposure to DMMP, the peak at 1690 cm^–1^ disappears as the benzoic acids are no longer present as dimers due to occupation of these sites by hydrogen-bonded DMMP.^43^ The vibration peaks at approximately 910, 825, and 780 cm^–1^ appear after exposure to DMMP, representing the symmetrically stretched P–O–C bonds in DMMP. The peaks’ appearance confirms the presence of hydrogen-bonded DMMP in the polymer.

It should be noted that the rate of absorption is ambiguous to determine as DMMP is unfit for a dynamic vapor sorption analysis, due to the low vapor pressure. The temperature during exposure controls the vapor pressure, which determines the evaporation rate of DMMP. Simultaneously, the temperature determines the polymer absorption rate as diffusion occurs more rapidly closer to the *T*_g_ (60 °C).^44,45^ The temperature influence on the evaporation and absorption of DMMP results in a clear difference in absorption over time for exposure at 25, 37, and 50 °C (Figure 2f). Similarly, the temperature influence on absorption is visible from the changes in the reflection spectra, although the occurrence of two peaks makes it ambiguous to quantify a peak shift (Figure S4). After absorption, the DMMP probably remains inside the polymer due to the limited diffusion through the surface layer having a higher *T*_g_ after releasing DMMP in combination with the slow evaporation kinetics.

**Figure 3:**
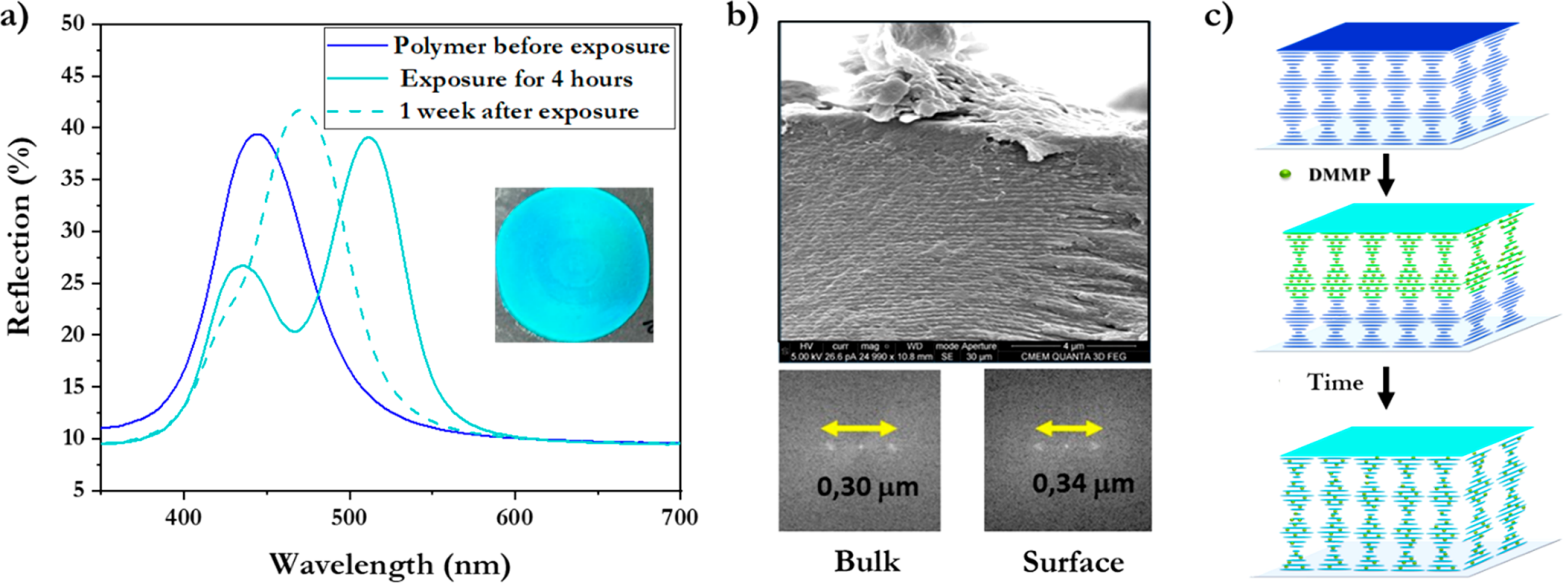
Kinetics and diffusion model of absorption at 37 °C. (a) UV–vis reflection spectra after exposure to DMMP vapor. Inset is a photograph of the sample after the 4 h exposure. (b) Cross-sectional SEM image of the polymer immediately after exposure to DMMP vapor, with insets showing a two-dimensional Fourier transformation to illustrate the pitch in the cholesteric order. (c) Schematic representation of DMMP vapor absorption, causing a change in the pitch of the helical structure through a diffusion front, which distributes throughout the polymer over time.

The optical response as a function of absorbed DMMP concentration of an unsaturated photonic polymer obtained by 4 h exposure at 37 °C was studied by UV–vis spectroscopy (Figure 3). Remarkably, two separate peaks are observed immediately after exposure, resulting in 6.1 wt % absorption of DMMP in the polymer: there is a decrease of reflectance intensity at 436 nm, and a new peak appears around 512 nm (Figure 3a). The color changes in this range can also be seen by the naked eye, transitioning from an initial dark blue to cyan after exposure. Cross-sectional SEM measurement of the polymer reveals two regions, each with their own helical pitch. A pitch length of 0.30 μm was measured at the non-exposed side that corresponds to the reflection band of 436 nm, indicating an unaltered helical pitch. In the region close to the surface that was exposed to DMMP, the pitch length increased to 0.34 μm, corresponding to reflection at 512 nm (Figure 3b). These results confirm the formation of a sharp boundary between swollen and unswollen polymeric regions. These data suggest the possible absorption mechanism: as DMMP vapor molecules begin diffusing into the polymer, they form hydrogen bonds with the existing hydrogen bond network between benzoic acid units.^32^ This results in an increase of the pitch through a diffusion front that progresses from the surface layers of the material into deeper layers that are unaltered. In the non-Fickian diffusion model, the absorption of vapors could be promoted by the local increase of guest molecules’ concentration, accompanied by a softening of the material. Indeed, a decrease of the glass transition temperature from 60 to −2 °C for the polymer absorbed with DMMP was measured with DSC (Figure S2). Although it is difficult to decouple the rate of absorption from the rate of DMMP evaporation, the diffusion front after 4 h of exposure indicates that the rate of absorption is rather slow; otherwise, the absorption would be more dispersed throughout the polymer. This temperature effect displays that a larger difference between the polymer temperature and the *T*_g_ makes it more difficult for the diffusion front to penetrate throughout the polymer.

When the polymer exposed for 4 h at 37 °C was remeasured after 1 week, the reflection peaks were merged into one peak, averaging the two previous peaks (Figure 3a). Optically, the effective color did not obviously change and remained cyan, whereas TGA of the polymer indicated the same 6.1 wt % DMMP was still absorbed. This implies that the absorbed DMMP is more evenly distributed throughout the polymer (Figure 3c).

The photonic polymer serves as an optical indicator, as the reflection spectrum of an exposed polymer is correlated to the quantity of molecules absorbed. The linear correlation between the wavelength shift of the optical response and the absorbed quantity of DMMP suggests that 1 wt % of DMMP absorption is detectable as a 4 nm wavelength shift (Figure S5). Such a shift could be detected with optical spectroscopy and optoelectronic sensors. This means, as an estimate of sensitivity, that a concentration of 2 ppm is detectable by 1 mg of photonic polymer. However, slow diffusion combined with the low sensitivity of the response exceeds the requirements for active protection. For decontamination, the optical feature allows the amount of absorption to be indicated, and robotic cleanup devices can read out the absorption status.

**Figure 4:**
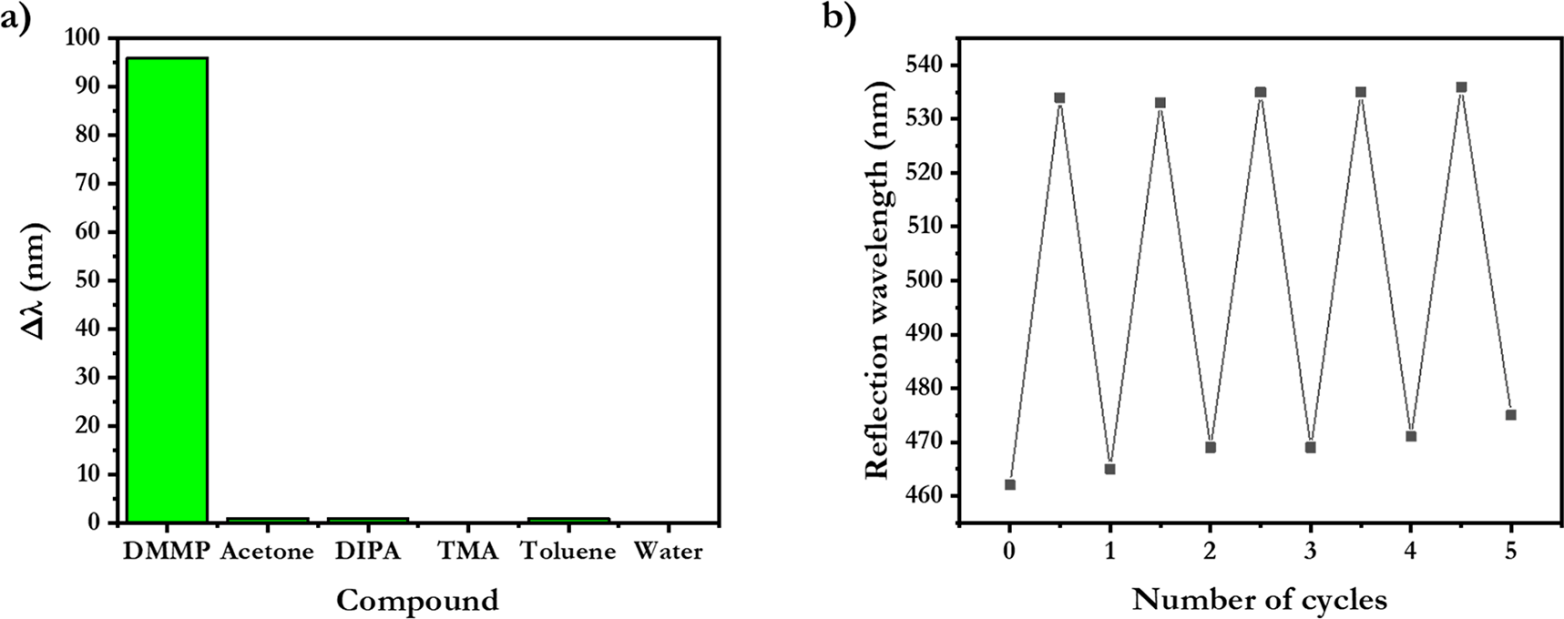
Polymer absorption performance characteristics. (a) Selectivity measured by the wavelength shift after exposure to vapors of volatile liquids: acetone, DIPA, TMA, toluene, and water. (b) Repeatability demonstrated by five cycles of absorption and release of DMMP.

Selectivity of the photonic polymer response toward DMMP was tested by exposing the CLC photonic polymer to vapor concentrations of several volatile liquids (20 μL at 37 °C), including water and Lewis base analytes such as diisopropylamine (DIPA) and trimethylamine (TMA). Previously, we reported on a similar polymer demonstrating an optical response to a high concentration of trimethylamine.^38^ This resulted in a loss of the selective reflection peak but not to a color change. Therefore, the reflection band shift induced by the swelling of the polymer is a selective feature. Monitoring selective reflection wavelength changes after the interaction with the vapor molecules of selected analytes displays the selectivity for organophosphate, confirming the possible application of these CLC photonic polymers for sensing applications of organophosphate compounds (Figure 4a). The phosphoryl oxygen results in a strong interaction hydrogen bond with the acid. Moreover, our photonic absorbent polymer has free volume created by the evaporation of MAA that allows for a volumetric selectivity for molecules with a phosphoryl oxygen that have a molecular volume smaller than that of MAA, which is true for a select number of nerve agents and pesticides.

The absorbed DMMP can be quickly released by heating the CLC absorbent to *T* = 140 °C, implying that the process is repeatable. Therefore, the reactivation cycles of the sensor were tested, which shows a high reversibility of the SRB shift over at least five absorption and release cycles (Figure 4b).

## Conclusion and Outlook

This work demonstrates a photonic liquid crystal polymer material simultaneously able to both absorb and detect DMMP vapor, a proxy for Sarin nerve agent. The polymer was able to absorb and retain the vapor from the atmosphere, causing a volume change and a visible color shift in the reflected wavelength.

Thermogravimetric analysis shows the ability of the material to absorb 22.7% of its weight in DMMP vapor. This absorption capacity is related to weight fraction of porogen used to prepare the responsive photonic polymer. The diffusion mechanism of gas molecules, observing a deviation from Fick’s model, reveals the decisive role of the polymers’ glass transition temperature. Supramolecular interactions combined with a specific volumetric porosity provides selectivity toward DMMP over competing atmospheric contaminants. Moreover, the optical response of the polymer to different amounts of DMMP was demonstrated: as an estimate of sensitivity, 2 ppm DMMP is detectable by 1 mg of photonic polymer. The material features vapor absorption without releasing it in the environment unless the film is heated. Unlike most of the reported sensors for organophosphate chemical warfare agents, the CLC polymer studied in this work is also reusable for several reactivation cycles.

A CLC polymer able to simultaneously identify, detect, and reversibly retain a nerve agent simulant from the vapor phase with selectivity for phosphoryl oxygen has not been reported before. Easy readout, portability in the field, and the lack of activation steps contribute to ease of use, suggesting CLC polymers are promising materials for several applications in national security, environmental monitoring, and protection against the most hazardous known gas compounds. These systems have the potential to be a remediation tool for indoor environments, such as buildings, or as protective packaging for safer transport and stockpile during decontamination procedures of hazardous compounds by providing detection and absorption of spillage and evaporation. Improvement of the absorption behavior is possible by implementing more hydrogen bonding sites and a greater fraction of porogens. However, this acts in opposition to the optical detection sensitivity. The sensitivity of detection can be improved by lowering the glass transition temperature of the polymer, allowing for faster diffusion and easier expansion of the polymer. This can be achieved through a decrease in the cross-link density by lowering the amount of diacrylate molecules or by making use of a chain extender such as a dithiol or secondary amine. Furthermore, lowering the amount of porogen decreases the absorption limit of the system and indirectly increases the sensitivity. Lastly, the amount of polymer used as the device is correlated to the amount of DMMP that needs to be absorbed. There is an inevitable trade-off between the absorption capacity and the optical response of the absorbent polymer.

## Materials and Methods

### Materials

2-Methyl-1,4-phenylene bis(4-(3-(acryloyloxy)propoxy)benzoate) [RM 257] (**1**) and 4-methoxyphenyl 4-((6-(acryloyloxy)hexyl)oxy)benzoate [RM 105] (**2**) were obtained from Merck. 4-((6-(Acryloyloxy)hexyl)oxy)benzoic acid (**3**) and 4-((6-(acryloyloxy)hexyl)oxy)-2-methylbenzoic acid (**4**) were purchased from Synthon. Irgacure 369 (**6**) was purchased from CIBA. Chiral dopant (*R*)-(+)-3-methyladipic acid (**5**) and dimethylmethylphosphonate (**7**) were from Sigma-Aldrich, as well as trimethylamine and diisopropylamine. Solvents tetrahydrofuran and toluene were purchased from Biosolve, and acetone was purchased from Sigma-Aldrich.

### Characterization

The reflection of the CLC polymers was measured through ultraviolet–visible spectroscopy using a PerkinElmer LAMBDA 750 with a 150 mm integrating sphere over a range of 400–750 nm. A Varian 670 FT-IR spectrometer with slide-on ATR (Ge) was used to record IR spectra. Thermogravimetric analysis was performed in a TA TGA Q500 with a constant heating rate of 5 °C/min. Thermal transitions of the liquid-crystalline polymers were analyzed by differential scanning calorimetry using a TA Instruments Q2000 calorimeter with constant heating and cooling rates of 10 °C/min.

### Functionalization of Glass Slides

Glass slides were first cleaned by sonication (2-propanol, 30 min), followed by treatment in a UV–ozone photoreactor (Ultra Violet Products, PR-100, 20 min) to activate the glass surfaces. The glass surfaces were then modified by spin-coating (3000 rpm, 45 s) with 3-(trimethoxysilyl)propyl methacrylate solution (1 vol % solution in a 1:1 water–isopropyl alcohol mixture) or 1*H*,1*H*,2*H*,2*H*-perfluorodecyltriethoxysilane solution (1 vol % solution in ethanol) to obtain methacrylate-functionalized and fluorinated alkylsilane-functionalized glass substrates, respectively, followed by curing (100 °C, 10 min).

### Preparation of CLC Polymer Film

The CLC mixture of 200 mg consisting of 22.55 wt % of RM 257 (**1**), 27.70 wt % of RM 105 (**2**), 16.40 wt % of both 6OBA (**3**) and 6OBAM (**4**), (*R*)-(+)-3-methyladipic acid (**5**), and 1.5 wt % of Irgacure 369 (**6**) was dissolved in tetrahydrofuran (1 mL). One hundred microliters of this solution was dropped on a methacrylate-functionalized 3 × 3 cm glass slide. After the solvent was evaporated by heating at 55 °C, a perfluoro-coated 3 × 3 cm glass slide was placed directly on top and cooled to room temperature while simultaneously shearing along one direction to obtain a planarly aligned green color film. It was then photopolymerized by shining UV light (48 mW/cm^–2^ intensity in the range of 320–390 nm) for 5 min, after which the upper perfluoro-coated glass was removed. Photopolymerization was carried out with an Omnicure series 2000 EXFO lamp. The absorbent polymer was obtained by heating the pristine film at 200 °C for 45 min.

#### Detection of DMMP

The detection test was set up by placing the polymer film on the glass substrate in a 0.2 L vial in an oven to have the whole system isothermal. After equilibration, a specific volume of liquid DMMP was spread on the walls followed by immediately closing the vial. After exposure, the sensor was removed and the UV–vis spectrum was recorded, followed by TGA analysis.

#### Calculation of the DMMP Volumes Needed for Saturation

Based on the ideal gas law and the vapor pressure of DMMP, *n*/*V* = *p*/*RT*. The molar amount of gas can be calculated for a liquid volume. Additionally, the ratio of the molar amount of DMMP vapor and air gives a ppm level at saturation.

#### Sample Preparation for Scanning Electron Microscopy Measurements

The cholesteric structure was analyzed by scanning electron microscopy using a Quanta 3D FEG, and the polymer was cryogenically broken in liquid nitrogen to obtain a cross section and sputter-coated with gold at 60 mA over 30 s. The settings for SEM analysis in secondary electron mode were acceleration of 5 kV, working distance of 10 mm, and under high vacuum.

#### Helical Pitch Calculation

To calculate the pitches of the polymer films partly absorbed and fully absorbed with DMMP, the following equation was used: λ = *n* × *P* × cos(θ), where λ is the Bragg reflection wavelength or selective reflection band, *n* is the average refractive index (1.5) of the mixture, and θ is the angle of the incident light.

## Supporting Information Available

TGA spectra to demonstrate evaporation (Figure S1); DSC spectra indicating polymer glass transition temperatures (Figure S2); TGA spectra of a DMMP absorbed polymer film immediately after exposure and after 10 weeks storage at room temperature (Figure S3); UV–vis reflection spectra of the photonic polymer exposed at different temperatures and times (Figure S4); UV–vis reflection shift of the photonic polymer film as a function of absorbed DMMP with a linear fit to the data (Figure S5) (PDF)

## Abbreviations

CLC: cholesteric liquid crystal
CWA: chemical warfare agent
DMMP: dimethyl methylphosphonate
DSC: differential scanning calorimetry
MAA: (*R*)-3-methyladipic acid
SEM: scanning electron microscopy
SRB: selective reflection band
TGA: thermogravimetric analysis
