# Supplementary material for: Photonic Liquid Crystal Polymer Absorbent for Immobilization and Detection of Gaseous Nerve Agent Simulants
Source: ACS Appl Opt Mater. 2022 Sep 6;1(1):107–14. doi: 10.1021/acsaom.2c00014 (PMC9903360; doi:10.1021/acsaom.2c00014)
Supplement: Supplementary file 1 — ot2c00014_si_001.pdf [file ot2c00014_si_001.pdf]

Supporting information

A photonic liquid crystal polymer absorbent for immobilization and detection of gaseous nerve agent simulants

Yari Foelen,[a,d] Roberta Puglisi,[b] Michael G. Debije,[a,d] Albert P. H. J. Schenning[a,c,d,]*

[a] Laboratory of Stimuli-responsive Functional Materials and Devices, Department of Chemical Engineering and Chemistry, Eindhoven University of Technology, Den Dolech 2, 5600MB Eindhoven, The Netherlands.

[b] Department of Chemical Sciences, University of Catania, Viale A. Doria 6, 95100 Catania, Italy.

[c] SCNU-TUE Joint Laboratory of Device Integrated Responsive Materials (DIRM), South China Normal University, Guangzhou Higher Education Mega Center, 510006, Guangzhou, China.

[d] Institute for Complex Molecular Systems, Eindhoven University of Technology, Den Dolech 2,

5600 MB, Eindhoven, The Netherlands.

Corresponding Author

Albert P.H.J. Schenning, a.p.h.j.schenning@tue.nl, Department of Chemical Engineering and Chemistry, Eindhoven University of Technology, Den Dolech 2, 5600, MB Eindhoven, The Netherlands.

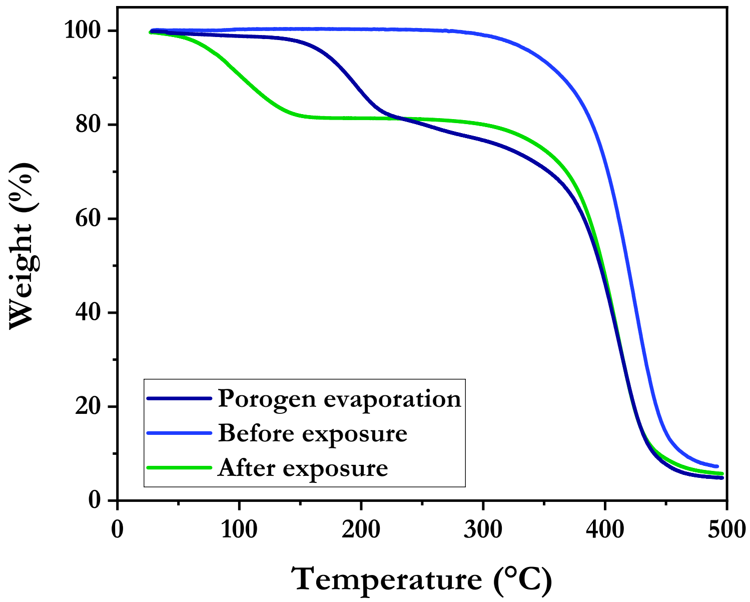

Figure S1. TGA spectra demonstrate the weight loss induced by evaporation of the porogen, the polymer degradation, and the total absorption capacity as weight loss during heating of the DMMP absorbed polymer.

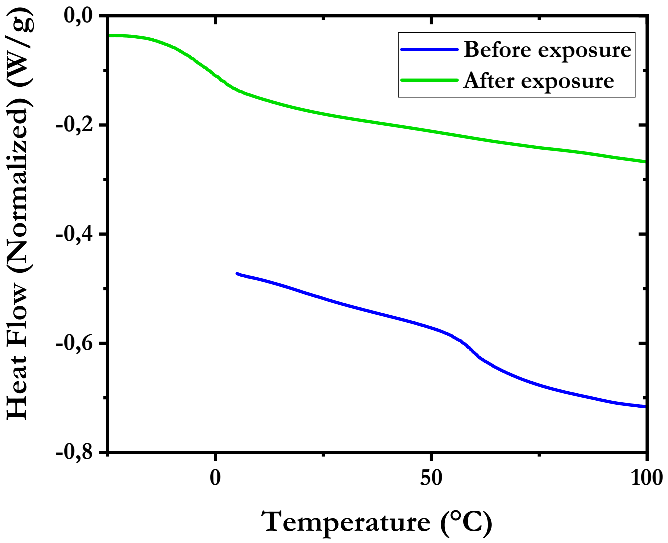

Figure S2. DSC spectra before and after 4 hours exposure to DMMP at 37°C, indicating a lowering of the glass transition temperature of the photonic polymer.

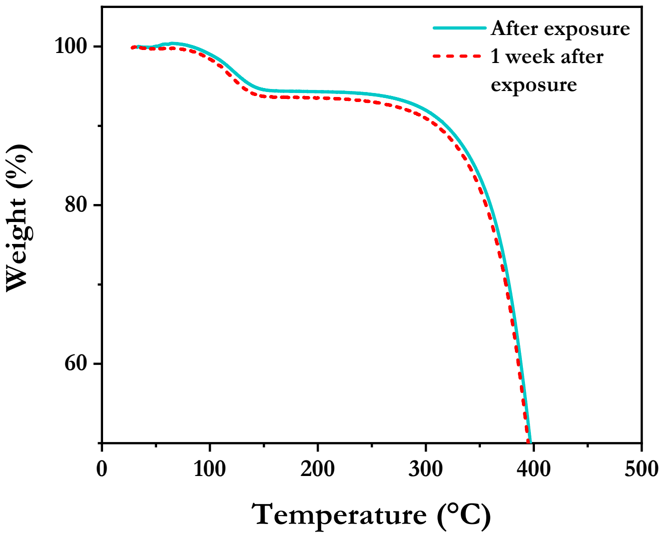

Figure S3. TGA spectra of a DMMP absorbed photonic polymer film immediately after exposure and after ten weeks storage at room temperature, showing no loss in the amount DMMP absorbed.

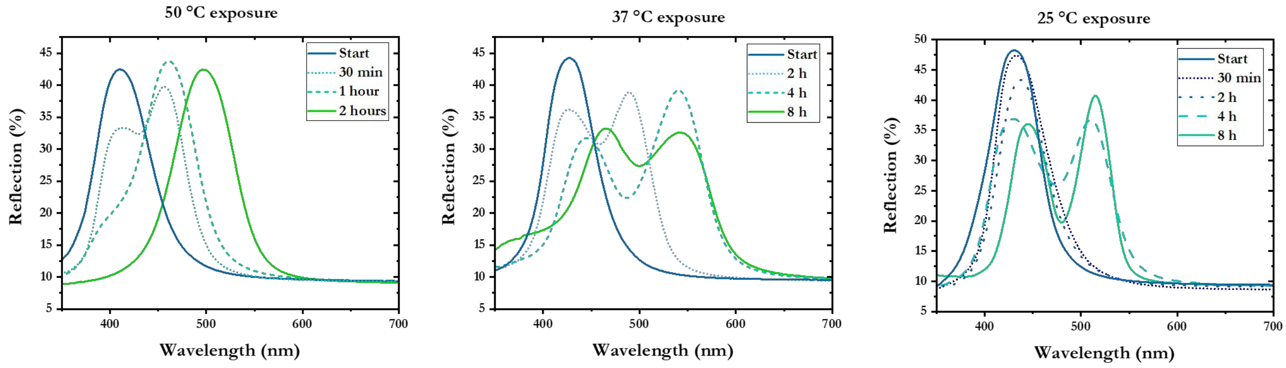

Figure S4. UV vis reflection spectra of the photonic polymer exposed at different temperatures and times, corresponding to the absorption measurements determined by TGA data in figure 2b.

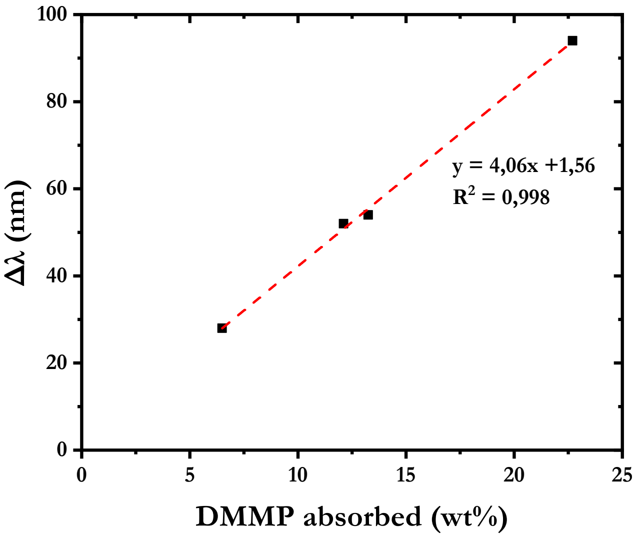

Figure S5. UV vis reflection shift of the photonic polymer film as a function of absorbed DMMP with a linear fit to the data.
